# Impact of CYP24A1 overexpression on growth of colorectal tumour xenografts in mice fed with vitamin D and soy

**DOI:** 10.1002/ijc.29717

**Published:** 2015-08-17

**Authors:** Julia Höbaus, Samawansha Tennakoon, Petra Heffeter, Charlotte Groeschel, Abhishek Aggarwal, Doris M. Hummel, Ursula Thiem, Rodrig Marculescu, Walter Berger, Enikö Kállay

**Affiliations:** ^1^ Department of Pathophysiology and Allergy ResearchMedical University of ViennaViennaAustria; ^2^ Department of Medicine IInstitute of Cancer Research and Comprehensive Cancer Center of the Medical University, Medical University ViennaViennaAustria; ^3^ Research Platform ‘Translational Cancer Therapy Research’ViennaAustria; ^4^ Division of Nephrology and Dialysis, Department of Medicine IIIMedical University of ViennaViennaAustria; ^5^ Department of Laboratory MedicineMedical University of ViennaViennaAustria; ^6^ Present address: Department of Pediatrics/Endocrinology, Stanford University School of MedicineStanfordCA

**Keywords:** colon cancer, vitamin D, CYP24A1, soy, xenograft

## Abstract

Our previous studies showed that the 1,25‐dihydroxyvitamin D (1,25‐D_3_) catabolizing enzyme, 1,25‐dihydoxyvitamin D 24 hydroxylase (CYP24A1) was overexpressed in colorectal tumours and its level correlated with increased proliferation. We hypothesised that cells overexpressing CYP24A1 have growth advantage and a diet rich in vitamin D and soy would restore sensitivity to the anti‐tumourigenic effects of vitamin D. Soy contains genistein, a natural CYP24A1 inhibitor. To determine causality between CYP24A1 and tumour growth, we established xenografts in male SCID mice with HT29 cells stably overexpressing either GFP‐tagged CYP24A1 or GFP. Mice were fed with either high (2500 IU D_3_/kg) or low vitamin D (100 IU D_3_/kg) diet in the presence or absence of soy (20% diet). *In vitro,* cells overexpressing CYP24A1 grew faster than controls. 1,25‐D_3_, the active vitamin D metabolite, reduced cell number only in the presence of the CYP24A1 inhibitor VID400. Regardless of the amount of vitamin D in the diet, xenografts overexpressing CYP24A1 grew faster, were heavier and more aggressive. Soy reduced tumour volume only in the control xenografts, while the tumours overexpressing CYP24A1 were larger in the presence of dietary soy. In conclusion, we demonstrate that CYP24A1 overexpression results in increased aggressiveness and proliferative potential of colorectal tumours. Irrespective of the dietary vitamin D_3_, dietary soy is able to increase tumour volume when tumours overexpress CYP24A1, suggesting that combination of vitamin D_3_ and soy could have an anti‐tumourigenic effect only if CYP24A1 levels are normal.

## Abstract 2

**What's new?**

Vitamin D_3_ supplementation is linked to reduced tumour growth in the colon, but colorectal tumours may also overexpress the vitamin‐D‐degrading enzyme CYP24A1, potentially undermining the benefits of vitamin D intake. We show that in a mouse xenograft model, CYP24A1 overexpression confers growth advantage to colorectal tumours, regardless of vitamin D intake. Combination of dietary vitamin D and soy increases volume and weight of tumours overexpressing CYP24A1. The findings warrant exploration of the effects of specific CYP24A1 inhibitors in CYP24A1‐overexpressing colorectal tumours.

**Abbreviations**
1,25‐D_3_: 1,25‐dihydroxyvitamin D_3_
15‐PGDH: 15‐hydroxyprostaglandin dehydrogenase
25‐D_3_: 25‐hydroxyvitamin D_3_
CDC6: cell division cycle 6
CRC: colorectal cancer
CMV: cytomegalovirus
COX‐2: cyclooxygenase‐2
CYP24A1: 1,25‐dihyroxyvitamin D‐24 hydroxylase
DAPI: 4′,6‐diamidino‐2‐phenylindole
DMEM: Dulbecco's modified eagle's medium
ER: estrogen receptor
FCS: fetal bovine serum
GFP: green fluorescence protein
MCM2: minichromosome maintenance complex component 2
PBS: phosphate buffered saline
PBST: 1X phosphate buffered saline with 0.01% tween‐20
RXR: retinoid X receptor; SXR, steroid and xenobiotic receptor
VDR: vitamin D receptor

The prohormone vitamin D_3_ is biologically inert and requires two hydroxylation steps in the liver and kidney to form the physiologically active 1,25‐dihydroxyvitamin D_3_ (1,25‐D_3_).^1^ 1,25‐D_3_ is a key player in the regulation of calcium homeostasis and bone mineralization. In recent years, the nonclassical functions of 1,25‐D_3_ have been studied extensively and include effects on proliferation, angiogenesis, differentiation, and apoptosis.^2^, ^3^ Many cell types express vitamin D system genes,^4^ being able to synthesise 1,25‐D_3_ from its precursor 25‐hydroxyvitamin D_3_ (25‐D_3_) locally. Colonocytes express the vitamin D receptor (VDR), the vitamin D‐metabolizing enzyme CYP27B1 as well as the vitamin D‐degrading enzyme, 1,25‐dihyroxyvitamin D‐24 hydroxylase (CYP24A 1).^5^, ^6^ The latter is an inner mitochondrial membrane enzyme^7^ and is a 1,25‐D_3_ target gene. Its expression is rapidly increased in the presence of 1,25‐D_3._ 8

Epidemiological studies suggest increased colorectal cancer (CRC) risk in patients with low blood 25‐D_3_ levels.^9^, ^10^ Several animal models demonstrate that serum vitamin D_3_ level is a key determinant for the development and progression of colon cancer and dietary vitamin D_3_ supplementation is important for suppression of intestinal tumourigenesis.^11^ We demonstrated recently that vitamin D_3_ supplementation alone is able to reduce the severity and number of chemically induced preneoplastic lesions in the colon of mice.^12^ Feldman and colleagues demonstrated that both vitamin D supplementation as well as 1,25‐D_3_ injections efficiently reduced growth of both breast and prostate xenografts in mice.^13^

CYP24A1 levels are upregulated in various solid tumours including colorectal tumours.^14^, ^15^, ^16^ Increased expression of CYP24A1 is an early event during colorectal carcinogenesis and the upregulation is already observed in colonic adenomas.^17^ The high CYP24A1 expression in tumour cells probably leads to depletion of local 1,25‐D_3_ levels in the tumour environment, decreasing its antitumourigenic functions. Recently, we observed a strong correlation between expression of CYP24A1 and the expression of proliferation markers in colorectal tumours suggesting that high CYP24A1 levels may provide a proliferative advantage to tumours.^17^, ^18^

If there is a causal link between CYP24A1 expression and tumour proliferation, tumours overexpressing CYP24A1 would grow faster and a selective inhibition of CYP24A1 in the tumour combined with increased vitamin D supplementation could reduce tumour growth. Soy protein contains high levels of the isoflavone genistein,^19^ which acts as a CYP24A1 inhibitor.^20^, ^21^ Genistein was already shown to inhibit growth in colonic adenomas and carcinomas^22^, ^23^ and combination of 1,25‐D_3_ with genistein resulted in synergistic growth inhibition of primary prostate epithelial cells.^24^ Thus, we hypothesized that adding soy to a vitamin D rich diet could reduce xenograft growth by inhibiting CYP24A1 function and thereby prolonging the half‐life of vitamin D metabolites.

In this study, we evaluated the causality between CYP24A1 overexpression and increased proliferation. We established xenografts in male SCID mice using a colon cancer cell line stably overexpressing CYP24A1. Mice were fed with low (100 IU D_3_/kg diet) or high (2500 IU D_3_/kg diet) vitamin D_3_ to study the effect of dietary vitamin D supplementation on xenograft growth. Additionally, half of the animals received soy protein (20% diet, instead of casein) containing the natural CYP24A1 inhibitor genistein. We observed increased tumour aggressiveness and larger tumours in CYP24A1‐overexpressing xenografts compared with controls. Interestingly, dietary soy increased volume and weight of tumours overexpressing CYP24A1.

## Material and Methods

### Generation of stable cell lines

HT29 cells were seeded (1.5 × 10^5^ cells/well) in 24‐well plates and cultured in complete Dulbecco's Modified Eagle Medium (DMEM) growth medium containing 10% FBS, 100 U/ml penicillin/streptomycin, 10 mM hepes and 2 mM l‐glutamine (Life Technologies) at 37°C in a humidified atmosphere of 5% CO_2_. At 50%–80% confluency, cells were transfected with either the pCMV‐CYP24A1‐GFP or pCMV‐GFP vector (OriGene Technologies) harbouring a G418 resistance gene, using Lipofectamine LTX^TM^ reagent (Invitrogen) according to the manufacturer's protocol. After the first passage, transfected cells were seeded in six‐well plates, and selection was performed by culturing the cells in culture medium containing G418 (Sigma Aldrich, USA). Cells were cultured for more than one month to select for stably transfected clones. The selected colonies were screened for the expression of either GFP (HT29^GFP^) or CYP24A1‐GFP (HT29^CYP24A1‐GFP^) and cells were sorted using a cell sorter system (MoFlo Astrios, Beckman Coulter) based on GFP expression. During culture maintenance, cells were treated with G418 to maintain selection pressure. However, experiments were performed in the absence of G418 to allow comparison with untransfected HT29 cells.

### *In vitro* treatments

HT29, HT29^GFP^ and HT29^CYP24A1‐GFP^ cells were cultured in DMEM medium containing 10% FBS. Before 1,25‐D_3_ treatment, the medium was replaced with serum‐free complete growth medium supplemented with 5 mg/ml insulin, 5 mg/ml transferrin and 5 ng/ml sodium selenite (ITS, Life Technologies) overnight. Cells were treated with 10 nM or 100 nM 1,25‐D_3_ for 6 hrs or 24 hrs. Ethanol‐treated cells were used as controls.

### Immunofluorescence staining of colon cancer cells

HT29, HT29^GFP^ and HT29^CYP24A1‐GFP^ cells were seeded on sterile glass coverslips and treated with 100 nM 1,25‐D_3_ for 20 hrs. Post treatment, cells were fixed in 3% paraformaldehyde, permeabilized using 0.2% Triton‐X (Sigma Aldrich, USA) in phosphate‐buffered saline (PBS) for 20 min and blocked with 10% goat serum (Jackson ImmunoResearch, USA) in PBS. Cells were incubated with CYP24A1 primary antibody (1:100, SC66851, Abcam, Cambridge, UK) in 5% goat serum in PBS, for 1 hr. Rabbit IgG (Abcam, Cambridge, UK) was used as negative control. Dylight 549 goat‐anti‐rabbit IgG (1:500, Vector Laboratories, UK) was used as secondary antibody. Nuclei were stained with DAPI (Roche, Vienna, Austria) for 10 min and mounted with Fluoromount‐G (Southern Biotech, USA). Images were acquired using TissueFAXS (TissueGnostics, Vienna, Austria). Determination of the localization of the CYP24A1 was achieved by performing a colocalization staining with a mitochondrial tracker, MitoTracker RedCMXRos (Invitrogen, Grand Island, NY, USA) in HT29^CYP24A1‐GFP^ cells.

### Growth rate of colon cancer cells

HT29^CYP24A1‐GFP^ and HT29^GFP^ cells were seeded in 96‐well plates. The cells were treated for 14 days with 10 nM 1,25‐D_3_ in the presence and absence of the CYP24A1 inhibitor VID400 (200 nM) or 25 µM genistein (Sigma Aldrich, USA); viable cells were counted with T10 Automated Cell counter (BioRaD, CA).

### Animals, diet and establishment of xenograft

Six weeks old male C.B‐17/lcrHan^®^Hsd‐Prkdc*^scid^* (severe combined immunodeficient) mice (Harlan Laboratories) were housed in the animal facility at the Institute of Cancer Research of the Medical University of Vienna under controlled environment with 12 hrs light–dark cycle. Guidelines of the European Union Regulations on Care and Use of Laboratory Animals were followed when maintaining the living conditions and performing the experiments. The study was approved by the Ethics Committee of the Medical University of Vienna as well as the local authorities (Nr: BMWF‐66.009/0115‐II/3b/2013).

Upon arrival, animals were grouped into four diet groups (10 mice/group) and received a modified AIN‐93G diet containing either low vitamin D_3_ levels (100 IU D_3_/kg diet), 100 IU D_3_/kg vitamin D_3_ diet together with 20% soy protein, high vitamin D_3_ diet (2500 IU D_3_/kg diet), or 2500 IU D_3_/kg vitamin D_3_ diet together with 20% soy, all from Ssniff (Ssniff Special Diet GmBH, Germany).

Forty animals were maintained on the above diets for 2 weeks. Xenografts were established by injecting 1 × 10^6^ cancer cells into the left flank of the animals, in each diet group (*n* = 10): half of the mice were injected with HT29^CYP24A1‐GFP^ cells (*n* = 5), the other half with HT29^GFP^ cells (*n* = 5) (Fig. 3). Xenograft growth was monitored by tracking the tumour size, tumour weight and body weight of the animals. Three weeks after the xenograft injection, the tumours in some animals started to penetrate the skin. Therefore, all mice were sacrificed by cervical dislocation. The whole experiment lasted for 5 weeks (35 days): 2 weeks acclimatization plus 3 weeks after xenograft inoculation. Liver, lung, tumour and kidney were removed immediately and shock frozen in liquid nitrogen. Blood was collected for the plasma analysis of calcium and 25‐D_3_.

### Plasma parameters

Plasma calcium concentrations were measured photometrically by the NM‐BAPTA method using the Cobas Ca2 (Calcium Gen.2) clinical diagnostic test on a Cobas c 702 clinical chemistry analyser (Roche Diagnostics, Indianapolis, IN, USA). Maximal coefficient variation of the test was 2.5%. 25‐D levels were determined with the Liaison 25 OH Vitamin D Total Assay on a Liaison clinical immunology analyser (Diasorin, Saluggia, Italy).

### VDR immunofluorescence staining of xenografts

Staining of the paraffin‐embedded tissue was performed as previously described.^18^ Staining for the VDR was performed using VDR primary antibody (1:200, Sigma Aldrich SAB 4503071) in 0.05% PBST. Expression of CYP24A1 in xenografts were determined by using a turboGFP antibody (1:100, Origene TA150041) in 0.05% PBST to detect the tagged GFP. Whole tissue slide images were acquired using TissueFAXS (TissueGnostics, Vienna, Austria).

### Tissue samples, RNA isolation, reverse transcription and quantitative RT‐PCR

Frozen tissues were homogenised with TRIzol reagent (Invitrogen, Grand Island, NY, USA) using Precellys ceramic beads (2.8 mm) in a Precellys 24‐Dual homogenizer at 6000rmp for 20 sec two times as previously described.^25^ RNA isolation, reverse transcription and qRT‐PCR analysis were performed as described previously.^18^ Mean of three genes, B_2_M, RPLP0, QARS were used for normalization of gene expression in the xenograft tissue, EEF1B2 was used for the kidney and Beta2‐microglobulin in the cell lines. All values were set relative to the calibrator, obtained from commercially available total RNA (Clontech, USA), and are presented as 2^−ΔΔCT^. Primer sequences are described in Supporting Information Table T1.

### Statistical analysis

SPSS version 22 (IBM, USA) was used to perform all statistical analyses and GraphPad Prism version 6 (GraphPad Software Inc., USA) was used for illustration. Data were log transformed to achieve normal distribution, where necessary. Comparison between two groups were performed by unpaired *t* test whereas, two‐way ANOVA followed by Tukey's multiple correction was used to compare more than two groups. Area under the curve was used to compare effects between groups in tumour volume.

## Results

### Stable overexpression of CYP24A1 in HT29 cells

To study the impact of CYP24A1 overexpression *in vitro*, we established a stable cell line overexpressing CYP24A1 under a constitutive, strong cytomegalovirus (CMV) promoter. We transfected the colon cancer cell line HT29 either with GFP‐tagged CYP24A1 or an empty vector containing only GFP. Several clones were generated and all had similar levels of CYP24A1. A representative CYP24A1‐GFP clone was selected (HT29^CYP24A1‐GFP^) and compared with the control cell line transfected only with the plasmid containing GFP (HT29^GFP^) and with the parental HT29 cell line. The same clone was used also in the *in vivo* experiments.

**Figure 1:**
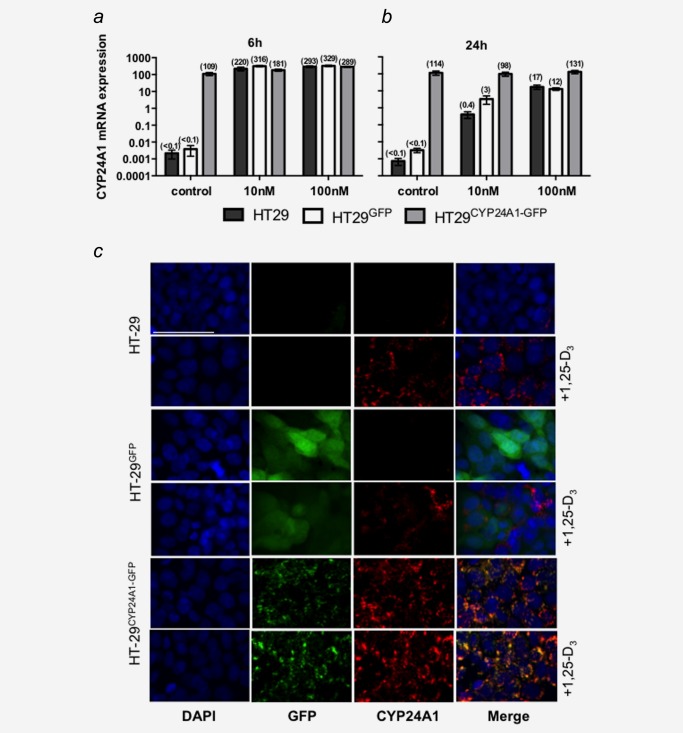
mRNA and protein expression of CYP24A1 in cell lines. The parental cell line (HT29) was stably transfected with a GFP‐tagged plasmid (HT29^GFP^) or a plasmid encoding for the fusion protein CYP24A1‐GFP (HT29^CYP24A1‐GFP^). Cells were treated for 6 h (***a***) or 24 h (***b***) with 10 nM 1,25‐D_3_, 100 nM 1,25‐D_3_ or vehicle control. mRNA levels of CYP24A1 were assessed by real time qRT‐PCR. All values were set relative to total RNA calibrator and are presented as 2^−ΔΔCT^. Bars represent mean ± SEM of three independent experiments. (***c)*** Cells were stained for CYP24A1 expression on coverslips after 20 h of treatment with 100 nM 1,25‐D_3_, or vehicle. Vector GFP expression is detectable in HT29^GFP^ and HT29^CYP24A1‐GFP^ cells but is absent in the parental cell line. Basal CYP24A1 (red) protein expression is low with exception of HT29^CYP24A1‐GFP^ cells but is inducible with 1,25‐D_3_. White line represents 50 μm.

HT29 cells are characterized by low basal expression of CYP24A1, which is highly inducible upon treatment with 1,25‐D_3_. On mRNA level, basal expression of CYP24A1 was very low in HT29^GFP^ cells and the parental cell line HT29, while it was significantly higher in the CYP24A1‐GFP‐transfected cells (Figs. 1 *a* and 1 *b*). Treatment with 10 nM and 100 nM 1,25‐D_3_ for 6 hrs resulted in a strong induction of basal CYP24A1 in both HT29 and HT29^GFP^ cells. In HT29^CYP24A1‐GFP^ cells the increase is only marginal and is comparable with CYP24A1 levels in control cells after 6 hrs 1,25‐D3 treatment (Fig. 1 *a*). This indicates that the constitutive expression of CYP24A1 in HT29^CYP24A1‐GFP^ does not exceed physiological levels of CYP24A1 expression reached after treatment with 1,25‐D_3_. After 24 hrs of 1,25‐D_3_ induction, the CYP24A1 mRNA expression levels decreased in the parental cells and in HT29^GFP^ cells but remained high in HT29^CYP24A1‐GFP^ cells (Fig. 1 *b*).

To assess translation of CYP24A1 mRNA to protein in HT29^CYP24A1‐GFP^ cells, we performed immunofluorescence staining of CYP24A1 before and after treatment of cells with 100 nM 1,25‐D_3_. In HT29 and HT29^GFP^ cells, CYP24A1 expression was almost undetectable in the vehicle control group and increased after 20 hrs of 1,25‐D_3_ treatment (Fig. 1 *c*). As expected, HT29^CYP24A1‐GFP^ cells showed strong CYP24A1 protein expression basally and the CYP24A1 staining overlapped with GFP expression as typical for fusion proteins. CYP24A1 staining was observed only in the cytoplasm in a localized and punctated manner (Fig. 1 *c*). The staining performed with the mitochondrial marker (MitoTracker RedCMXRos) confirmed that the CYP24A1 is localized in the mitochondria (Supporting Information Fig. S1).

### CYP24A1 overexpression reduces the antiproliferative function of 1,25‐D_3_ *in vitro*

**Figure 2:**
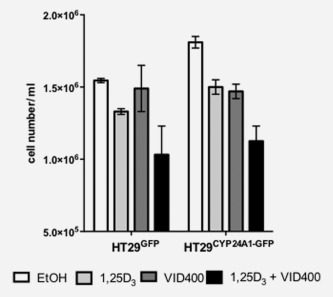
Response of stably transfected cells to treatments of 1,25‐D_3_ and CYP24A1 inhibitor. Cell number was assessed after 14 days of treatment with 10 nM 1,25‐D_3_ in combination with the CYP24A1 inhibitor VID400 (200 nM). Bars, vehicle control (white bar), 10 nM 1,25D_3_ (light grey bar), 200 nM VID400 (dark grey bar) and combination of 1,25D_3_ with VID400 (black bar) represent mean ± SEM of two independent experiments in triplicates.

To test whether the CYP24A1 overexpression is functional, growth rates of these cells were determined. 1,25‐D_3_ has been previously shown to exert antiproliferative effects in various cancer cell lines including HT29.^26^ *In vitro*, number of cells/ml was increased in HT29^CYP24A1‐GFP^ cells when compared with HT29^GFP^ cells (Fig. 2). Treatment of cells with 10 nM 1,25‐D_3_ reduced cell number in both cell lines when compared with their respective vehicle control. The CYP24A1 inhibitor VID400^27^ (200 nM) reduced the number of HT29^CYP24A1‐GFP^ cells to control levels (HT29^GFP^), while it had no effect on growth of HT29^GFP^ cells. Combination of 1,25‐D_3_ and VID400 had a stronger effect in HT29^CYP24A1‐GFP^ cells than in the control HT29^GFP^ cells.

### Effects of vitamin D_3_ and soy supplementation on plasma levels of 25‐D_3_, calcium and renal vitamin D metabolism

**Figure 3:**
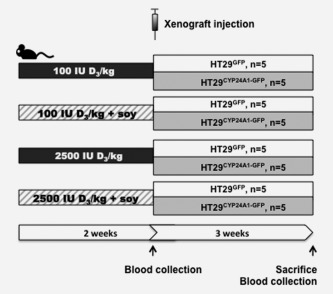
Schematic presentation of the mouse model. Mice were divided into four groups receiving low (100 IU D_3_/kg diet) or high (2500 IU D_3_/kg diet) vitamin D diet ± soy (20%). After 2 weeks HT29^GFP^ or HT29^CYP24A1‐GFP^ cells (10^6^ per mouse) were injected to the right flank of the mice. The experiment was terminated at the end of the 5^th^ week from the starting of the experiment (2 weeks acclimatization to the diet plus 3 weeks of xenograft growth).

Six‐week old male SCID mice were divided into four feeding regimes (100 IU D_3_/kg vitamin D_3_ diet, 100 IU D_3_/kg vitamin D_3_ diet together with 20% soy protein, 2,500 IU D_3_/kg vitamin D_3_ diet, 2,500 IU D_3_/kg vitamin D_3_ diet together with 20% soy), with 10 mice/group. Animals were maintained on the specific diet for 2 weeks after which HT29^CYP24A1‐GFP^ cells were inoculated to half of the animals (five mice each) from all diet groups. The other half received HT29^GFP^ cells. Tumour growth was monitored until the end of the experiment (Fig. 3).

**Figure 4:**
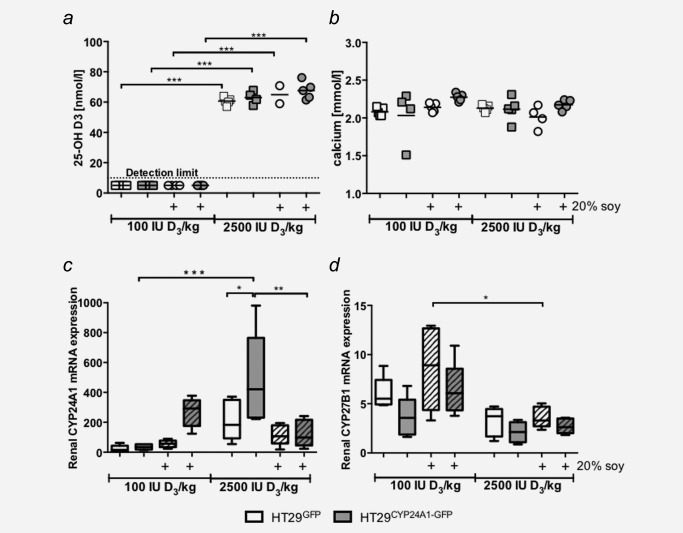
Plasma 25‐OH D_3_, calcium levels and renal mRNA expression. (*a)* Plasma 25‐OH D_3_ levels of the mice receiving 100 IU D_3_/kg diet at the end of the experiment were below the lower detection limit of the assay (10 nmol/l), whereas 2,500 IU D_3_/kg diet increased plasma levels above 50 nmol/l, horizontal lines indicate mean. (*b*) Plasma calcium levels did not change between high and low vitamin D diet at the end of the experiment. (*c,d*) Renal mRNA expression of the 1,25‐D_3_ degrading enzyme CYP24A1 and the 1,25‐D_3_ activating enzyme CYP27B1 were determined by real time qRT‐PCR. Patterned bars: diet containing 20% soy. Whiskers represent min to max. Two‐way ANOVA with Tukey's multiple comparison, *p < 0.05, **p < 0.01 ***p < 0.001 was used to determine the statistical significance.

After the 2 weeks acclimatization to the diet, at the time of the xenograft inoculation, in the low vitamin D group the plasma 25‐D_3_ levels were already decreased (29.7 ± 4.2 nmol/l, data not shown) while at the end of the experiment the 25‐D_3_ levels were below detection limit (<10 nmol/l) (Fig. 4 *a*). In the group receiving high vitamin D diet plasma 25‐D_3_ levels reached 64.6 ± 4.16 nmol/l already after 2 weeks (data not shown) and this level has not changed during the following 3 weeks of tumour growth (63.96 ± 5.25 nmol/l at the end of the experiment), indicating that peak levels are already reached within 2 weeks and dietary supplementation does not increase plasma 25‐D_3_ levels further (Fig. 4 *a*). Before xenograft injection, we did not observe significant differences in plasma calcium levels among the diet groups 2.06 ± 0.07 mmol/l (data not shown) and calcium levels remained stable (Fig. 4 *b*). Thus, no hypercalcaemia was observed in this experiment.

Surprisingly, we observed differences in renal CYP24A1 expression between HT29^CYP24A1‐GFP^ and HT29^GFP^ xenograft bearing mice, which was dependent on the diet. In addition, CYP27B1 expression tended to be lower in HT29^CYP24A1‐GFP^ xenograft bearing mice. These observations suggest that the cellular composition of the xenograft influences renal vitamin D metabolism.

In response to high vitamin D_3_ diet renal mRNA expression of CYP24A1 increased when compared with the levels in mice receiving low vitamin D_3_. This increase was higher in mice bearing CYP24A1‐overexpressing tumours (*p* < 0.001, Fig. 4 *c*). Interestingly, addition of soy to the low vitamin D_3_ diet increased renal expression of the 1,25‐D catabolizing enzyme, CYP24A1 in HT29^CYP24A1‐GFP^ xenograft‐bearing mice. Whereas soy supplementation in the high vitamin D_3_ diet group reversed this effect (*p* < 0.01, Fig. 4 *c*).

Overall renal CYP27B1 mRNA expression levels are lower in the high vitamin D_3_ diet group when compared with the low vitamin D_3_ group. This difference reached significance (*p* < 0.05) in GFP bearing xenografts receiving high vitamin D_3_ and soy (Fig. 4 *d*).

### Effects of CYP24A1 levels and experimental diet on tumour behaviour

**Figure 5:**
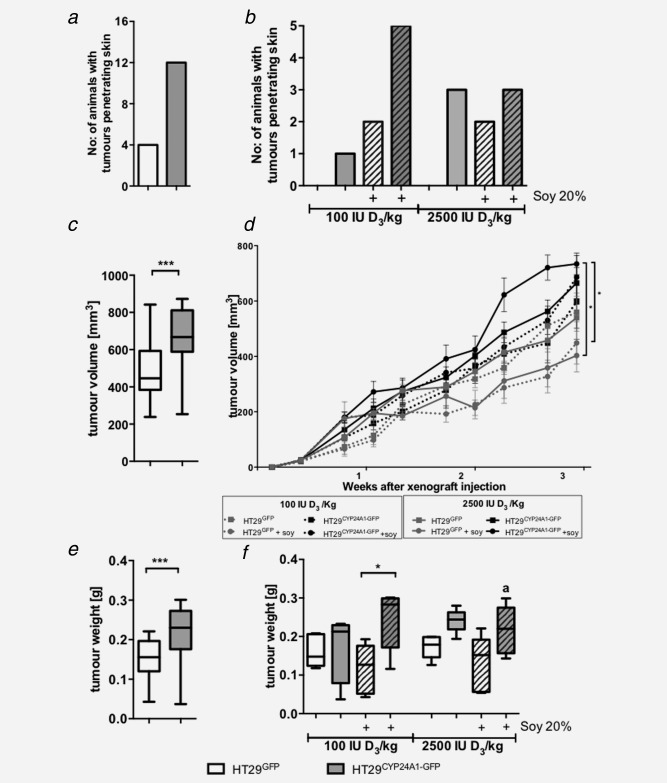
Tumour penetration, growth curve and weight of xenografts in male SCID mice. *(a)* Number of animals with tumours penetrating the skin; effect of CYP24A1 expression (*n* = 20 per group). (*b*) Tumour penetration per group (*n* = 5) at the end of the experiment. (*c*) Effect of CYP24A1 expression on tumour volume (*n* = 20 per group). Tumour volume was calculated using the formula (length × width^2^)/2. (*d*) Effect of diet on tumour volume per group (*n* = 5). HT29^GFP^ xenografts: grey line, HT29^CYP24A1^ xenografts: black line, low vitamin D diet: dashed lines, high vitamin D diet: continuous lines, supplemented with soy: lines with circles, absence of soy: lines with squares. Area under the curve was used for the analysis. (*e*) Effect of CYP24A1 expression on tumour weight (*n* = 20 per group). (*f*) Tumour weight per group (*n* = 5) is shown in the different diet groups. As three out of five animals had bite marks on the tumours, the weight of CYP24A1 overexpressing tumours in the high vitamin D and soy group is lower than expected (*a*). Data points represent mean ± SEM. Patterned bars: diet containing 20% soy. Whiskers represent min to max. Statistical significance was determined using unpaired *t*‐test (*a, c, e*), area under the curve (*d*) and two‐way ANOVA with Tukey's multiple comparison (*b, d, f*), *p < 0.05, ***p < 0.001.

When tumours started lacerating the skin in some animals, all animals were sacrificed and the experiment was terminated. HT29^CYP24A1‐GFP^ xenografts penetrated the skin earlier than the control xenografts irrespective of the given diet (Fig. 5 *a*). CYP24A1 overexpression resulted in the most aggressive xenografts under low vitamin D_3_ diet supplemented with soy (Fig. 5 *b*).

Combining all diet groups, the tumours overexpressing CYP24A1 were significantly larger (*p* < 0.001, Fig. 5 *c*) and heavier (*p* < 0.001, Fig. 5 *e*).

Analysing the effect of the diet and CYP24A1 level on tumour volume (Fig. 5 *d*), we observed that in general, CYP24A1 overexpression (black lines) increased tumour volume when compared with the HT29^GFP^ xenografts (grey lines), independent of diet. Dietary vitamin D_3_ had no significant effect on the growth of HT29^GFP^ xenografts. However, the volume of CYP24A1 overexpressing xenografts in the high vitamin D group is larger than in the low vitamin D group. Dietary soy reduced the tumour volume in GFP bearing xenografts (grey lines with circles), but increased that of CYP24A1 overexpressing xenografts (black lines with circles). Significance was reached only between CYP24A1 overexpressing tumours *vs*. HT29^GFP^ xenografts receiving high vitamin D with soy.

CYP24A1 overexpression increased tumour weight (Fig. 5 *f*) when compared with the HT29^GFP^ xenografts, except in the mice receiving low vitamin D diet without soy. Dietary vitamin D_3_ (100 IU/kg *vs* 2500 IU/kg) had no significant effect on tumour weight. Dietary soy had no effect in GFP bearing xenografts while it increased the tumour weight when CYP24A1 expression was high. CYP24A1‐overexpressing xenografts in mice receiving high vitamin D_3_ diet supplemented with soy were chewed (a). Therefore, tumour weight in this group is smaller than it would have been, had the tumours stayed intact (Fig. 5 *f*). Additionally we would like to note that not only the size but also the density of the tissue affects the weight of the tumour.

Neither the diet nor CYP24A1 overexpression in the xenografts affected significantly the body weight of the mice (Supporting Information Fig. S2).

### Expression of the vitamin D system genes and vitamin D target genes in the xenografts

**Figure 6:**
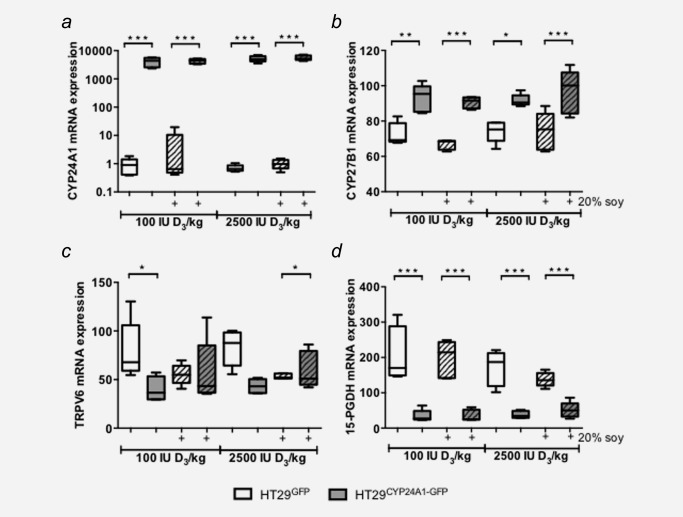
Expression of vitamin D system genes and vitamin D target genes in xenografts. mRNA levels were determined by qRT‐PCR as described in the methods section. (*a*) mRNA expression of the 1,25‐D_3_‐degrading enzyme CYP24A1. (*b*) mRNA expression of the 1,25‐D_3_‐activating enzyme CYP27B1. Expression of vitamin D target genes: (*c*) the calcium channel TRPV6, and (*d*) prostaglandin E2‐degrading enzyme 15‐PGDH. Patterned bars: diet containing 20% soy. Data were log transformed to achieve normal distribution and statistical analysis was performed using two‐way ANOVA with Tukey's multiple comparison, *p < 0.05, **p < 0.01, ***p < 0.001.

To test whether CYP24A1 expression was still present in the HT29^CYP24A1‐GFP^ xenografts after 3 weeks *in vivo*, we analysed mRNA expression in the tumours at the end of the experiment. In all xenografts generated from HT29^CYP24A1‐GFP^ cells, CYP24A1 mRNA expression was on average more than 2500‐fold higher than in the xenografts generated from HT29^GFP^ cells, irrespective of the diet (Fig. 6 *a*). Staining of the xenografts with t‐GFP, as this detects both the GFP tag in the control group and the fusion protein in the CYP24A1‐overexpressing group demonstrated that the fusion protein CYP24A1‐GFP was still expressed after 3 weeks of growth *in vivo* (data not shown). Moreover, overexpression of CYP24A1 in HT29^CYP24A1‐GFP^ xenografts was accompanied by an increased mRNA expression of CYP27B1, indicating a possible elevated 1α‐hydroxylation to compensate for rapid 1,25‐D_3_ degradation (Figs. 6 *a* and 6 *b*). mRNA expression patterns were similar between high and low vitamin D_3_ diet groups for all investigated vitamin D target genes. Expression of the 1,25‐D_3_ target gene TRPV6, a calcium channel, did not change with increased vitamin D_3_ diet (Fig. 6 *c*). The prostaglandin dehydrogenase 15‐PGDH, the major prostaglandin‐catalyzing enzyme was downregulated in HT29^CYP24A1‐GFP^ xenografts compared with HT29^GFP^ xenografts, irrespective of the diet (Fig. 6 *d*). We did not observe significant differences in mRNA expression of the other investigated genes by dietary soy either.

## Discussion

CYP24A1 overexpression is observed in various solid tumours, 18, 28, 29, 30, 31 indicating a selection advantage for tumour cells with high CYP24A1 expression. Recently, we found that colorectal tumours expressing high levels of CYP24A also expressed higher levels of proliferation markers.^18^ Therefore, to test whether there is a causal relationship between CYP24A1 overexpression and increased tumour growth, we compared xenografts overexpressing CYP24A1 with xenografts obtained from the same cells but with low basal CYP24A1 levels. Regardless of the diet, CYP24A1‐overexpressing xenografts grew faster and were more aggressive, as evidenced by more tumours penetrating the skin.

The cell line HT29^CYP24A1‐GFP^ constitutively expressed CYP24A1 at a level similar to that reached in HT29 cells stimulated with 1,25‐D_3_, indicating that overexpression yields levels of CYP24A1 mRNA that can be reached physiologically in a cell.

Recently, Tannour‐Louet *et al*. 28 demonstrated that stable transfection with shRNA against CYP24A1 attenuated xenograft growth of DU145 prostate cancer cells in combination with oral gavage of 1,25‐D_3_, while 1,25‐D_3_ alone only slightly reduced xenograft growth. Furthermore, Ki‐67 staining was significantly reduced in CYP24A1 shRNA xenografts under 1,25‐D_3_ administration, clearly showing that CYP24A1 expression abrogates the antiproliferative effects of 1,25‐D_3_. Our cell line data indicates that overexpression of CYP24A1 alone constitutes a proliferative advantage for these cells. In HT29^CYP24A1‐GFP^ cells a downregulation of the cell number was observed when the CYP24A1 inhibitor VID400 was administered in combination with vitamin D_3._ As VID400 alone was also able to reduce the growth potential of HT29^CYP24A1‐GFP^ cells, we speculate that CYP24A1 overexpression may affect cell growth also in a 1,25‐D_3_‐independent manner.

Dietary vitamin D_3_ is converted to 25‐D_3_ in the liver and released into the blood stream. Various vitamin D_3_ target tissues express CYP27B1 and can therefore metabolize 25‐D_3_ to 1,25‐D_3_, which then acts in an autocrine/paracrine‐manner.^1^ Increasing dietary vitamin D_3_ elevates the plasma 25‐D_3_ levels and thereby increases the amount of substrate for CYP27B1 at local sites including tumour xenografts. In our study, dietary vitamin D_3_ supplementation with 2500 IU D_3_/kg diet significantly (*p* < 0.001) raised plasma 25‐D_3_ levels to >60 nmol/l (63.96 ± 5.25) within 2 weeks, but has not increased further, suggesting that in these mice 2500 IU D_3_/kg vitamin D_3_ diet does not cause hypervitaminosis. As administration of vitamin D_3_ in its biologically inactive form has not increased systemic calcium levels; we could exclude hypercalcaemia as a side‐effect, which is often observed when administrating 1,25‐D_3._ 13 Local metabolism of 1,25‐D_3_ in extra‐renal tissues does not affect circulating levels of 25‐D_3._ 32 Thus we did not expect that overexpression of CYP24A1 in xenografts would affect systemic 25‐D_3_ levels. However, it seems that the tumours influenced the renal metabolism of 1,25‐D_3_ as CYP27B1 expression tended to be lower in the kidneys of mice harbouring CYP24A1‐overexpressing xenografts. Furthermore, CYP24A1 expression was significantly increased in the kidneys of mice harboring HT29^CYP24A1‐GFP^ xenografts and receiving high vitamin D_3_ diet, whereas soy supplementation reversed the effect. Thus, it appears that the xenografts affect also the renal vitamin D metabolism by paracrine mechanisms. However, as systemic 25‐D_3_ levels appear unaffected, the underlying processes are currently unknown and were beyond the scope of this study.

In general, CYP24A1 overexpressing xenografts were more aggressive and penetrated the skin earlier than control xenografts. The highest proportion of xenografts lacerating skin at the end of the experiment was observed in the low vitamin D_3_, high soy group. It is important to note that HT29^GFP^ xenografts caused skin laceration only if the diet contained soy but not in the absence of soy. At the same time, dietary soy delayed the growth of control xenografts, suggesting that tumour aggressiveness is independent of tumour volume. This is interesting as soy is proposed to reduce tumour growth in breast, prostate and colorectal cancer. Particularly the soy isoflavone genistein, has been shown to act as an anticancer agent through interfering with cell signalling, increasing apoptosis, decreasing the expression of androgen receptor and suppressing the prostaglandin pathway.^33^ Swami *et al*. observed an inhibition of proliferation in prostate epithelial cells *via* vitamin D signaling when treated with genistein in the presence of low doses of 1,25‐D_3._ 21 Arai *et al*.^34^ have shown that 10 µM genistein inhibited HT29 cell growth when the cells were treated for 96 hrs. Interestingly, treating the HT29^GFP^ and HT29^CYP24A1‐GFP^ cells with 25 μM genistein in the presence or absence of 10 nM 1,25D_3_ for 14 days caused a significant (*p* < 0.001) increase in cell number in the CYP24A1‐overexpressing cells but not in the HT29^GFP^ cells (Supporting Information Fig. S3). These *in vitro* data support our *in vivo* data that soy promotes the growth of the tumours overexpressing CYP24A1, regardless of vitamin D levels. There are reports showing that in some instances, soy or genistein did not have antiproliferative effect or even had reverse effects. A randomized controlled intervention study in patients diagnosed with adenomatous polyps showed no reduction in cell proliferation of the colorectal epithelium after a 12‐month dietary soy isoflavone intervention.^35^ Furthermore, in a patient‐derived prostate cancer xenograft model administration of genistein at low (80 mg/kg/day) as well as high (400 mg/kg/day) doses resulted in more metastases and in increased cell proliferation and decreased apoptosis in these metastases but not in primary tumours.^36^ This suggests that the activity of genistein is wider than just inhibition of CYP24A1. Since in our hands control xenografts grew slower under soy diet, but caused more skin infiltration, a similar mechanism as observed by Nakamura *et al* 36 may be involved. In this study the authors stated that the prostate cancer cell line (LTL163a) showed no expression of estrogen receptor (ER) alpha but had elevated levels of ER beta and reasoned that the effect of genistein is likely mediated by ER beta resulting in EGFR phosphorylation and increased proliferation.^36^ In addition, lower levels of estrogen could be causative for soy to act as a partial ER receptor agonist. As in our study expression of estrogen receptor alpha and beta was almost undetectable in all xenografts (data not shown), the observed effect of soy protein appears to be independent of ER signalling in the tumour. Furthermore, as mice received soy protein rather than genistein, we cannot conclude whether the observed adverse effect is caused by genistein or by other isoflavones found in soy.

The increase in CYP27B1 mRNA expression in the CYP24A1‐overexpressing xenografts indicate increased 1,25‐D_3_ synthesis in response to the rapid degradation. This is similar to reports in human CRC^18^ and breast cancer,^37^ where CYP24A1 overexpression goes hand in hand with higher CYP27B1 levels. However, similar to the human model, we reason that more than 2,500‐fold increase in CYP24A1 expression outweighs the moderate increase in CYP27B1 expression shifting the reaction towards 1,25‐D_3_ degradation. It is interesting that mRNA expression patterns of the vitamin D target genes studied appear to be influenced only by the level of CYP24A1 in the xenograft as well as soy supplementation. This is irrespective of dietary vitamin D_3_ levels as similar expression patterns are observed in the low and high vitamin D_3_ diet groups. In CYP24A1‐overexpressing xenografts, expression of 15‐PGDH is reduced compared with control xenografts. 15‐PGDH is the key prostaglandin catabolizing enzyme.^38^ Thus reduced expression may lead to higher prostaglandin levels in the xenografts further increasing angiogenesis and proliferation. In addition, we measured the expression of a number of other genes in the tumours including nuclear receptors (VDR, RXRα, SXR, ER α and β), cytochrome P_450_ enzyme (CYP3A4), proliferation marker (MCM2, CDC6, KI67), differentiation marker (sucrase isomaltase) and angiogenesis marker (CD31). Interestingly, in our study expression of these targets were not affected either by vitamin D_3_ or soy (data not shown).

1,25‐D_3_ signalling is mediated by VDR through binding to vitamin D‐responsive elements in promoters of target genes or distal enhancers. In colorectal cancer, VDR mRNA levels are decreased in the tumour tissue compared with the adjacent mucosa,^18^ while in breast cancer reports are contradictory.^37^, ^39^, ^40^, ^41^ Under high vitamin D_3_ diet, VDR protein expression was significantly (*p* < 0.01,) increased in tumours overexpressing CYP24A1 compared with control, and this effect was independent of dietary soy (Supporting Information Fig. S4).

In conclusion, here we provide evidence that the overexpression of CYP24A1 in a mouse xenograft model of colorectal cancer results in more aggressive tumours. While vitamin D_3_ alone had no significant effect on tumour growth, in combination with dietary soy increased tumour volume and weight when cells expressed elevated levels of CYP24A1. This suggests that targeted inhibition of CYP24A1 could be used as a possible additional therapy for colon cancer patients with tumours overexpressing CYP24A1, whereas high dietary vitamin D_3_ in combination with soy could be beneficial for prevention.

## Supporting information

Supporting Information

Supporting Information

Supporting Information

Supporting Information
