# Supplementary figures and images for: Impact of CYP24A1 overexpression on growth of colorectal tumour xenografts in mice fed with vitamin D and soy
Source: Int J Cancer. 2015 Aug 17;138(2):440–50. doi: 10.1002/ijc.29717 (PMC4832261; doi:10.1002/ijc.29717)

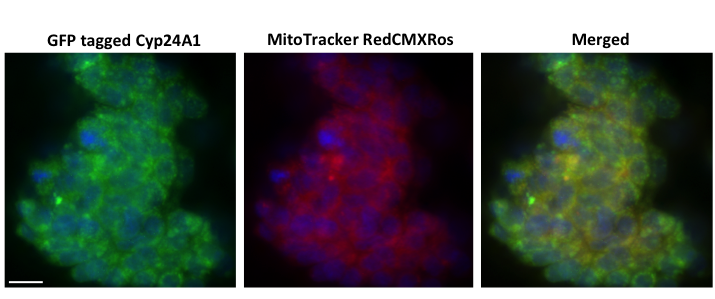

Supplement: Supplementary file 1 — Supporting Information [file IJC-138-440-s001.tif]

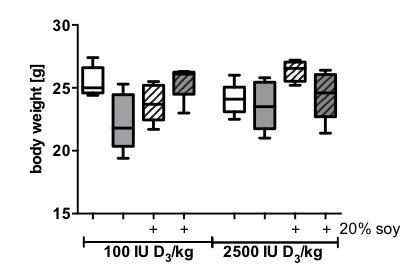

Supplement: Supplementary file 2 — Supporting Information [file IJC-138-440-s002.tif]

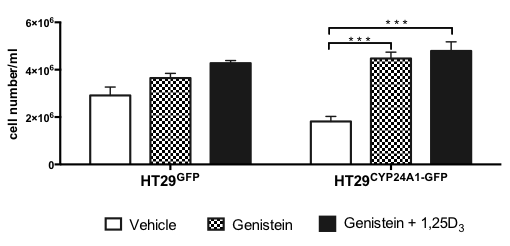

Supplement: Supplementary file 3 — Supporting Information [file IJC-138-440-s003.tif]

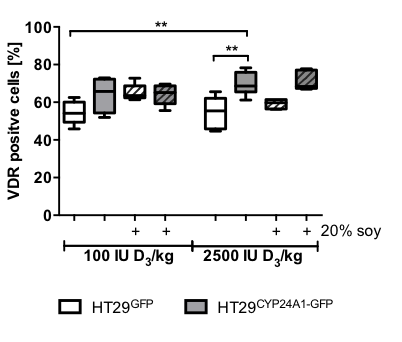

Supplement: Supplementary file 4 — Supporting Information [file IJC-138-440-s004.tif]
